# Predicting Tropical Dry Forest Successional Attributes from Space: Is the Key Hidden in Image Texture?

**DOI:** 10.1371/journal.pone.0030506

**Published:** 2012-02-20

**Authors:** J. Alberto Gallardo-Cruz, Jorge A. Meave, Edgar J. González, Edwin E. Lebrija-Trejos, Marco A. Romero-Romero, Eduardo A. Pérez-García, Rodrigo Gallardo-Cruz, José Luis Hernández-Stefanoni, Carlos Martorell

**Affiliations:** 1 Departamento de Ecología y Recursos Naturales, Facultad de Ciencias, Universidad Nacional Autónoma de México, Mexico City, Mexico; 2 Forest Ecology and Management Group, Wageningen University and Research Centre, Wageningen, The Netherlands; 3 Centro de Investigación Científica de Yucatán A.C., Mérida, Yucatán, Mexico; 4 Department of Plant Sciences, Faculty of Life Sciences, Tel Aviv University, Tel Aviv, Israel; Centre National de la Recherche Scientifique, France

## Abstract

Biodiversity conservation and ecosystem-service provision will increasingly depend on the existence of secondary vegetation. Our success in achieving these goals will be determined by our ability to accurately estimate the structure and diversity of such communities at broad geographic scales. We examined whether the texture (the spatial variation of the image elements) of very high-resolution satellite imagery can be used for this purpose. In 14 fallows of different ages and one mature forest stand in a seasonally dry tropical forest landscape, we estimated basal area, canopy cover, stem density, species richness, Shannon index, Simpson index, and canopy height. The first six attributes were also estimated for a subset comprising the tallest plants. We calculated 40 texture variables based on the red and the near infrared bands, and EVI and NDVI, and selected the best-fit linear models describing each vegetation attribute based on them. Basal area (*R* ^2^ = 0.93), vegetation height and cover (0.89), species richness (0.87), and stand age (0.85) were the best-described attributes by two-variable models. Cross validation showed that these models had a high predictive power, and most estimated vegetation attributes were highly accurate. The success of this simple method (a single image was used and the models were linear and included very few variables) rests on the principle that image texture reflects the internal heterogeneity of successional vegetation at the proper scale. The vegetation attributes best predicted by texture are relevant in the face of two of the gravest threats to biosphere integrity: climate change and biodiversity loss. By providing reliable basal area and fallow-age estimates, image-texture analysis allows for the assessment of carbon sequestration and diversity loss rates. New and exciting research avenues open by simplifying the analysis of the extent and complexity of successional vegetation through the spatial variation of its spectral information.

## Introduction

In the dawn of the 21st century the magnitude of the human footprint on the planet's ecological systems has become undeniable [1]–[4]. Although much emphasis has been placed on the effects of industrial activities and their potential contribution to global change through greenhouse gas emissions [5]–[8], the chronic effects of land clearance for the purpose of food production on the Earth's natural vegetation are likely to be among the most long-lasting human legacies [9]–[11]. Ecologists now acknowledge that the majority of the planet's vegetation during the present century will consist of secondary or successional communities: from now on we will co-exist with secondary forests, use them, and entirely depend on them [12], [13]. The maintenance of terrestrial biodiversity will be possible as long as we are capable of keeping expanses of secondary forests [14], [15], and the regulation of the world's ecosystems will be closely linked to their existence [13], [16]. Secondary forests have also been identified as important carbon reservoirs and may play a crucial role in mitigating future global warming [17]–[27].

Vegetation ecologists currently struggle in their attempts to distinguish secondary forests from primary vegetation through remote sensing [28]–[32]. More critical, however, is the difficulty in differentiating the various successional stages that secondary forests normally comprise and measure their extent [20], [33]–[37]. As their structure and functions depend on their succesional status, there is a strong need to efficiently evaluate the extent and complexity of secondary vegetation existing in any region and to discern its attributes.

Our success in achieving these goals will depend largely on our ability to estimate accurately the structure and diversity of secondary communities at broad geographic scales. Efforts to assess the extent of secondary vegetation and to distinguish its successional variants through remote sensing have followed several routes. Most studies estimating forest structure and diversity through satellite imagery have exclusively used image spectral features and their derived vegetation indices [20], [30], [34], [38]–[45]. There are, however, several problems related to this approach; for example, some remotely-sensed vegetation indices face the problem of saturation, i.e. they are unable to discriminate different plant communities beyond a certain biomass or canopy development threshold [22], [30], [39], [46], [47]. Moreover, although some studies have succeeded in discriminating forest successional stages accurately, they have been limited to the recognition of few broad stages, which do not reflect the continuous nature of the successional process [28], [34], [48]–[52].

Recent theoretical developments in Landscape Ecology have established the link between the structural and compositional complexity of vegetation and the spatial variability of its remotely-sensed signal [53]–[55]. This spatial variability is directly related to the heterogeneity of the plant community and can be assessed by analyzing the texture of a remotely-sensed image [33], [56]–[58]. Texture refers to the spatial variation of the elements of which any image is composed [59]. Although measures of texture have been commonly used as image descriptors in remote sensing analyses [46], [60], [61], the resolution of most sensors currently employed for this purpose has prevented the examination of the internal heterogeneity of plant communities, as the size of commonly-used pixels is too large to detect such small-scale variation [37], [62]. This drawback may be overcome by using very-high resolution imagery (VHR; pixels <10 m), currently available for most of the Earth's surface, as it provides a better match between pixel size and the internal variation of vegetation [33], [56]–[58], [63]–[66]. Therefore, we hypothesized that the textural information contained in VHR images has the potential to reflect the variability of secondary vegetation, allowing us to model the successional process.

The goal of this study was to examine the potential of textural properties of a VHR Quickbird image to model secondary vegetation attributes measured in the field, in a seasonally dry tropical region. We wanted to test the power of the texture of remote images to describe and predict vegetation attributes, while identifying those texture attributes with the highest predictive potential. In modeling the relationship between textural and vegetation attributes, we succeeded in producing simple models that can be easily obtained for many regions, and that have a straightforward biological interpretation.

## Materials and Methods

### Study area

**Figure 1:**
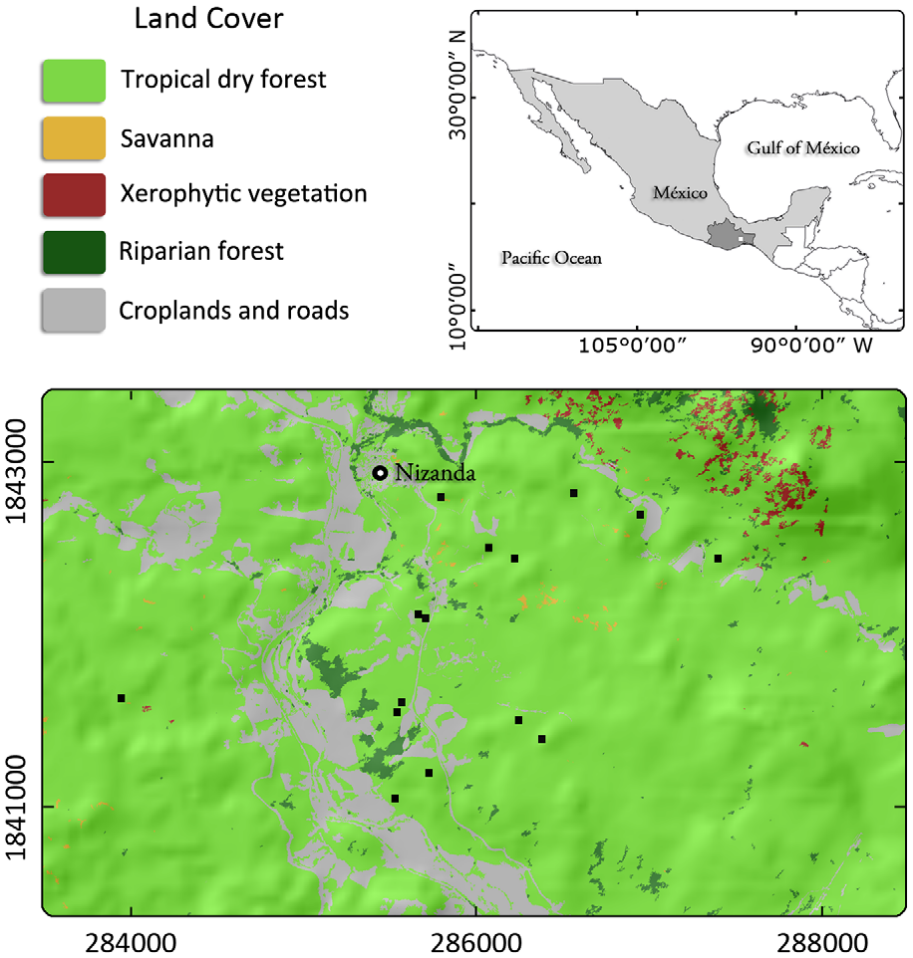
Study area (UTM zone 15n) and location of the secondary plots (▪) used for modeling their attributes from the texture derived from a Quickbird satellite image.

The study was conducted in the dry tropical region of Nizanda, Oaxaca State, Mexico (16°39.49′N, 95°0.66′W; Fig. 1). Mean annual temperature is 26°C and the total average annual rainfall is 900 mm, largely concentrated between June and October. The prevailing vegetation matrix is a low-stature (7–10 m) seasonally dry tropical forest [67], [68]. Traditional slash-and-burn agriculture is practiced in the area. Fields are typically cropped for one or two years before being abandoned [69], which results in a mosaic of differently-aged fallows spread across the area.

### Field data

Fourteen 30×30 m fallows with time since abandonment (age) ranging from 2 to ca. 60 years, and one mature forest site, were selected from field surveys conducted in 2005. Site age was obtained from interviews with landowners and verified through dendrochronology [70]. This set of fallows included a large range of environmental and vegetational heterogeneity [71], [72], from sites with a little dense canopy dominated by shrubs of open foliage, to sites with a dense plant cover and a low percentage of bare soil.

The sites were marked and designated as permanent sites in 2003 [69], [71], and detailed structural and floristic information was collected annually (Table 1 lists the variables used for vegetation description and their abbreviations). In each fallow, four 20×5 m transects (400 m^2^, subdivided in four 5×5 m quadrats) were established for the sampling of woody and succulent plants. In each transect, all individuals ≥5 cm DBH were sampled in the four quadrats; individuals with DBH≥2.5 cm but <5 cm were sampled in two quadrats, and individuals with ≥1 but <2.5 cm DBH were sampled in one quadrat only. For each individual, DBH and two orthogonal crown diameters (used to calculate crown areas) were measured. Structural variables were obtained by scaling the data to 1 ha. Based on this information, for each plot the _T_ and _U_ sets (i.e. Total and Upper, respectively) of structural and diversity attributes were prepared. The _T_ set included all sampled plants in the plot, whereas the _U_ set included only those plants that are more likely to be remotely sensed [73]. The _U_ set comprised those plants above the median in the frequency distribution of canopy cover; this subset represented between 50% and 75% of the basal area in a sites. For these two sets we calculated Dn (individuals in the sampled area), CC (the sum of the individual crown areas), BA (the sum of individual basal areas), S (number of species), and Simpson's *D*' and Shannon's *H*' diversity indices [74]. In addition, Hgt was calculated as the average of the heights of eight trees, each the tallest tree in the zones formed by two adjacent quadrats. Structural and diversity data for the study sites are shown in Table 2; please refer to Table S1 for information on within-site variability for those variables for which the calculation of such variation is feasible and sensible.

**Table 1:** Vegetational attributes used in the analysis and their abbreviations.

Vegetational attribute	Description	Abbreviation
Age	Time since abandonment of the fallow (years)	Age
Density	Individuals in the sampled area (individuals/ha)	Dn
Canopy cover	Sum of all individual crown areas by site (m^2^/ha)	CC
Basal area	Sum of all individual basal areas by site (m^2^/ha)	BA
Richness	Number of species	S
Height	Mean height (see Methods; m)	Hgt
Shannon's index	Diversity index (logits)	*H'*
Simpson's index	Diversity index (logits)	*D'*
Total set	All sampled plants	_T_
Upper set	Plants above the median of the CC cumulative	_U_

**Table 2:** Structural and diversity attribute values for 15 plots.

Age	Hgt	S_T_	S_U_	Dn_T_	Dn_U_	BA_T_	BA_U_	CC_T_	CC_U_	*H'*_T_	*H'*_U_	*D'*_T_	*D'*_U_
2	2.4	7	3	1850	40	1.023	0.808	4996.664	2954.275	1.325	0.518	0.394	0.728
3	2.7	5	4	4850	102	1.756	1.092	13929.704	7424.604	0.538	0.424	0.756	0.815
5	4.6	4	2	4750	66	6.526	3.887	18587.796	10168.898	0.571	0.136	0.734	0.940
7	4.7	6	1	1825	18	6.150	3.438	18949.464	9832.621	1.293	0	0.367	1
9	4.6	19	7	6775	91	11.068	6.341	31597.844	16515.046	1.975	0.954	0.245	0.539
12	6.1	15	5	4100	46	10.201	6.590	28930.809	14945.067	1.835	1.240	0.281	0.325
13	6.6	29	14	6475	82	15.344	10.523	32446.110	16544.336	2.561	1.851	0.139	0.264
18	7.3	17	10	6925	73	14.604	10.672	31694.175	15909.057	1.730	1.469	0.311	0.391
20	7.0	22	8	4425	58	14.234	8.744	29682.050	15220.037	2.250	1.387	0.220	0.358
25	6.4	12	5	3850	45	11.042	7.022	23283.665	12097.390	1.591	0.814	0.323	0.611
32	6.5	21	9	5600	70	15.464	10.401	33259.234	16699.067	2.299	1.404	0.162	0.372
38	6.5	41	28	7725	122	26.040	17.116	36110.813	18559.764	3.055	2.615	0.070	0.120
42	6.8	27	12	5550	99	21.611	11.820	32099.665	16774.770	2.720	1.640	0.093	0.278
60	8.3	36	11	4500	46	21.441	14.374	36176.727	18135.708	2.993	1.614	0.085	0.330
M	7.0	36	17	7675	57	29.641	15.329	42411.829	21630.317	3.019	2.314	0.071	0.147

See Table 1 for vegetational attributes abbreviations and units of measurement.

### Image processing

We used a high-resolution Quickbird satellite image (pixel size = 2.6 m) acquired in early December 2005. This date, which corresponds to the beginning of the dry season, was chosen to minimize cloud cover while ensuring the presence of foliage in the plants. The image was geometrically and atmospherically corrected to surface reflectance following Krause [75].

From the four available bands in this image, we selected the red (RED; 0.63–0.69 µm) and the near infrared (IR; 0.76–0.90 µm) [41], both of which are known to reflect the condition of vegetation functioning and overall condition. We also estimated two commonly used vegetation indices, namely the normalized difference vegetation index (NDVI) and the enhanced vegetation index (EVI) [47], both derived from the combination of the two bands, as follows:
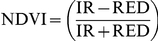


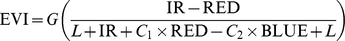
EVI incorporates empirical parameters (C_1_ = 6, C_2_ = 7.5) and the blue band (BLUE, 0.45–0.52 µm) for atmospheric correction, and sensitivity minimization of soil background reflectance variation (L = 1). The EVI does not saturate under dense canopy conditions as the NDVI does, and it also appears to be more sensitive to canopy structural characteristics [76].

### Image texture analysis

Image texture refers to the spatial variation and arrangement of the pixels of which any image is composed [59], [77]. Although this property can be extracted through a wide array of methods [77], [78], we chose to follow a statistical approach for this study. We calculated texture variables known as first-order and second-order measurements. First-order texture measures are statistical properties that do not consider pixel neighbor relationships and are derived from the original image values within a certain window (group of pixels); for this group of texture measures, the spectral variability within the window was assessed by calculating the range and the skewness of the values.

Unlike the textural variables from the first group, second-order measurements consider the spatial relations between groups of two neighboring pixels within the window [59], therefore these measurements were also selected because of their greater potential to reflect the heterogeneity in successional vegetation stands. The calculation of second-order variables involves the construction of Gray-Level Co-occurrence Matrices (GLCMs), which are matrices containing the probabilities of co-occurrence of pixel values for pairs of pixels in a given direction and distance. To construct such matrices we used a spatial distance of one pixel, four directions (0°, 45°, 90°, 135°), and 64 levels of gray. A co-occurrence matrix was constructed for each direction, and from each co-occurrence matrix a specific texture measurement was calculated for the window. The texture measurements of each direction were then averaged to obtain a single spatially-invariant texture value. This procedure was applied for the variables described in Table 3, which correspond to three groups of variables describing the degree of contrast between pixels (homogeneity, contrast, dissimilarity), the regularity in the pixels within a window (entropy, angular second moment), and the statistics derived from the GLCM (mean, variance, correlation).

**Table 3:** Texture variables derived from the grey-level co-occurrence matrix (GLCM).

Texture variable	Formula	Description
Mean	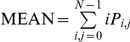	Mean of the probability values from the GLCM. It is directly related to the image spectral heterogeneity.
Variance	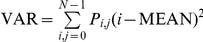	Measure of the global variation in the image. Large values denote high levels of spectral heterogeneity.
Correlation	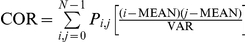	Measure of the linear dependency between neighbouring pixels.
Contrast	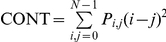	Quadratic measure of the local variation in the image. High values indicate large differences between neighbouring pixels.
Dissimilarity	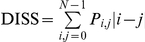	Linear measure of the local variation in the image.
Homogeneity	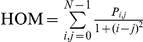	Measure of the uniformity of tones in the image. A concentration of high values along the GLCM diagonal denotes to a high homogeneity.
Angular second moment	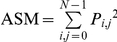	Measure of the order in the image. It is related to the energy required for arranging the elements in the system.
Entropy	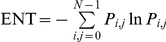	Measure of the disorder in the image. It is inversely related to ASM.

The abbreviations, formulas and descriptions of the eight texture variables used to model successional vegetation attributes are presented. *P_i_*_,*j*_ is the (*i*, *j*) element of the GLCM, and represents the probability of finding the reference pixel value *i* in combination with a neighbor pixel value *j*. Note that Σ*_i_*_,*j*_ *P_i_*_,*j*_ = 1.

The ten textural variables (two first-order and eight second-order variables) were calculated for the RED and the IR bands, as well as for the two vegetation indices (NDVI and EVI). We used a moving-window approach with a window size of 15 pixels to match the size of the sampling plots in the field; the central pixel value of this window was extracted from each of the 40 texture layers (four layers and ten variables). The entire procedure was programmed in the ENVI+IDL environment [79].

### Statistical analysis

We assessed the potential of the 40 texture variables to describe the observed changes in each of the 14 vegetation variables by means of linear models. In all cases the response variables were log-transformed before model fitting. This procedure guaranteed that the estimated values, when transformed to their original scale, would be positive; additionally, such transformation tends to homogenize the residuals, which are usually proportional to the mean in most probability density functions restricted to the positive numbers domain.

Assuming that different texture variables provide supplementary information about the remotely-sensed vegetation, we fitted three types of models, depending on whether they included one (560 models), two (10,920) or three (138,320) texture variables; no interaction was examined due to the limited degrees of freedom. For each of the three model types we selected the one having the largest coefficient of determination (*R* ^2^). For our analysis it was crucial to be able to compare the potential of textural variables to model different vegetational variables; for this purpose, *R* ^2^ was appropriate owing to its fixed range (from 0 to 1), unlike the commonly used Akaike's Information Criterion (AIC), whose range depends, among other things, on the sum of squares of the error of the dependent variable [80], making it impossible to compare models produced for different vegetational attributes.

Nevertheless, *R* ^2^ does not provide a good measure to determine whether, for any given vegetational variable, increasing the number of variables incorporated into the model improves its performance. Therefore, we compared the best-fit models of each type (i.e. with one, two, or three textural variables) through the small-sample-size bias-correction version of AIC (AICc); two models were considered to be equally good when the difference in AICc between them was <2 [80].

Due to the large number of models that were fitted and the small data set, it was expected that large *R* ^2^ values would be obtained by chance. To minimize this possibility, we produced null models by randomly sorting the texture- and vegetation-attribute data, fitting the same linear models as above, and selecting those with the largest *R* ^2^-values. This procedure was repeated 1,000 times and an empirical distribution of the largest expected *R* ^2^ was obtained. We then estimated the *P*-value associated with each model as the fraction of the simulated *R* ^2^ values that were greater than the observed ones. The median of the empirical distribution was used as a measure of the expected magnitude of *R* ^2^ under a completely random scenario.

To assess the predictive power of the models, we used leave-two-out cross-validation, i.e. we used a linear model fitted to the data from 13 plots (the calibration data) to predict the vegetation attribute of the remaining two plots (the validation data). We fitted a model for each possible split of the data set into calibration and validation subsets, and calculated the sum of squares between the estimated and observed values of the vegetation variable for the two validation plots. The model with the highest predictive power would be the one with the smallest average sum of squares (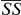) averaged over all the possible splits of the data set. We used a leave-two-out procedure to avoid the problems arising from the more popular leave-one-out approach [81]–[83], while keeping a sample size as large as possible for the estimation of the four parameters of the most complex models (those based on three texture variables).

The actual values of 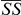 cannot be compared between vegetation attributes as they depend on the scale of measurement. A more informative statistic is given by the proportion of the variation of the vegetation attribute that can be predicted by the model. This statistic, expressed as a leave-*d*-out cross-validation *R* ^2^_CV_, is:
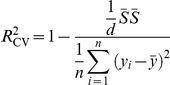
where *y_i_* is the vegetation attribute for the *i* ^th^ plot, and *n* is the number of plots. *R* ^2^_CV_ ranges from −∞ to 1, where a value of 1 means that a model predicts the validation data perfectly, while a negative value means that a model is over-fitted, because it would make worse predictions than those made by a null model. This statistic is dimensionless, so it can be used for comparing vegetation variables.

All statistical analyses were performed using R [84].

## Results

### Structural and diversity attributes of secondary vegetation

Two sets of structural and diversity attributes (_T_ and _U_) were analyzed for each sampled plot (see Table 2). For the _T_ set, which included all sampled plants in the plot, most structural variables exhibited clear increasing trends with successional development. However, this was not the case for Dn_T_, which did not show a clear successional pattern, and for *D*_T_ (dominance), which tended to decrease from young to old fallows. Some variables clearly showed stabilizing trends, particularly Hgt and CC_T_, or non-monotonic responses, as was the case of *S*_T_.

The _U_ set was arbitrarily defined as those trees that were above the median of the canopy cover cumulative distribution of each fallow. The proportion of BA_U_ with respect to BA_T_ was generally high, mostly above 60%, and often higher in mid-age fallows, despite the very low number of individuals included in this community's subset (in all cases less than 2% of stem density of the entire community).

### Descriptive models of successional vegetation attributes

We constructed three types of linear models to describe the relationship between the 14 vegetation attributes and the 40 image texture variables. These types corresponded to the number of texture variables used in constructing the model: one, two or three texture variables.

Most of the best fit models had relatively high significant *R* ^2^ values, and these increased as more variables were included. Among one-variable models, five out of 14 models had *R* ^2^>0.80, and this number increased to 10 and 12 for two- and three-variable models, respectively (Table 4). According to AICc, the latter were always the best-fit models (Table 4, TV entries in bold typeface). However, it must be noted that for half of the 14 vegetational variables the best two-variable models were equally good.

**Table 4:** Best descriptive linear models for 14 vegetation attributes (VA) as a function of 1, 2 and 3 textural variables (TV) with corresponding *R* ^2^, *P* and *R* ^2^_CV_ values.

VA	TV	*R* ^2^	*P*	*R* ^2^_cv_	IR_MEAN_	RED_VAR_	RED_MEAN_	NDVI_ASM_	EVI_ASM_	IR_CORR_	RED_DR_	NDVI_SKEW_	NDVI_MEAN_	EVI_MEAN_	NDVI_DISS_	RED_CONT_	IR_VAR_	EVI_SKEW_
BA_T_	1	0.802	<0.001	0.755		**−**												
	2	0.926	<0.001	0.900		**−**									**+**			
	**3**	0.958	<0.001	0.907		**−**	**−**									**+**		
BA_U_	1	0.784	0.001	0.733		**−**												
	2	0.916	<0.001	0.875		**−**									**+**			
	**3**	0.957	<0.001	0.906					**−**								**−**	**+**
Hgt	1	0.819	0.003	0.772		**−**												
	2	0.887	0.004	0.841		**−**												
	**3**	0.939	0.005	0.861	**−**				**−**									**+**
Age	1	0.822	<0.001	0.755			**−**											
	2	0.851	0.001	0.761			**−**											
	**3**	0.937	0.001	0.872			**−**				**−**					**+**		
CC_U_	1	0.802	0.001	0.629		**−**												
	2	0.884	0.002	0.777		**−**						**−**						
	**3**	0.931	0.002	0.807	**−**				**−**	**+**								
CC_T_	1	0.809	0.003	0.630		**−**												
	2	0.885	0.003	0.769		**−**						**−**						
	**3**	0.923	0.015	0.797	**−**				**−**	**+**								
S_U_	1	0.597	0.028	0.442	**−**													
	**2**	0.877	0.005	0.792	**−**			**−**										
	**3**	0.910	0.012	0.849	**−**			**−**									**+**	
S_T_	1	0.743	0.001	0.636			**−**											
	**2**	0.869	0.002	0.778	**−**													
	**3**	0.897	0.036	0.776	**−**			**−**				**+**						
*H'*_U_	1	0.603	0.019	0.464			**−**											
	2	0.820	0.012	0.721	**−**			**−**										
	**3**	0.881	0.062	0.678	**−**				**−**	**−**								
*D'*_T_	1	0.721	0.002	0.560			**+**											
	**2**	0.813	0.009	0.736									**−**	**+**				
	**3**	0.849	0.163	0.732				**+**					**−**	**+**				
*D'*_U_	1	0.573	0.049	0.423			**+**											
	**2**	0.774	0.035	0.650	**+**			**+**										
	**3**	0.843	0.178	0.436	**+**													
*H'*_T_	**1**	0.674	0.018	0.440			**−**											
	**2**	0.751	0.115	0.511						**−**	**−**							
	**3**	0.810	0.329	0.584						**−**	**−**							
Dn_U_	1	0.513	0.119	0.314														
	**2**	0.685	0.252	0.497									**−**					
	**3**	0.778	0.548	0.379								**−**	**−**					
Dn_T_	1	0.354	0.530	0.142				**−**										
	**2**	0.652	0.360	0.417					**−**					**−**				
	**3**	0.750	0.691	0.459	**−**	**−**					**+**							

Only those textural variables that were included in at least two models are shown. For the descriptive models conventional *R* ^2^ values are reported, while for the predictive models *R* ^2^_CV_ is reported, so the values are not strictly comparable (see Methods for explanation). *P*–values calculated from the empirical distribution of the largest expected *R* ^2^. TV entries in bold typeface are the best models according to AICc when comparing, for each VA separately, models of different type. The plus (+) and minus (−) symbols denote the sign of the coefficients in the models (values reported in Table S2). See Table 1 for vegetational attributes abbreviations. IR: near infra-red band, RED: red band, NDVI: Normalized Difference Vegetation Index, EVI: Enhanced Vegetation Index. See Table 3 for the description of textural variables denoted by subindices _MEAN_, _VAR_, _ASM_, _CORR_, _DR_, _SKEW_, _DISS_, and _CONT_.

**Figure 2:**
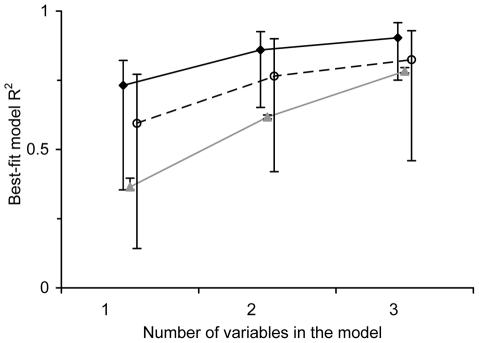
Fraction of the variation in vegetation attributes (median and range) explained by the descriptive (—♦—), predictive (- -○- -) and null (—▴—) models using a different number of textural attributes as explanatory variables. For the descriptive and null models conventional *R* ^2^ values are reported, while for the predictive models *R* ^2^_CV_ is reported, so the values are not strictly comparable (see Methods for explanation).

We measured the expected magnitude of *R* ^2^ under a completely random scenario through the median of its empirical distribution. The difference between this median and the observed one decreased as more variables were included, from 0.37 for models with one variable, to 0.24 and 0.11 in two- and three-variable models, respectively (Fig. 2). Also, the proportion of non-significant best-fit models increased from 0.14, through 0.21 to 0.43 as more variables were included. There were small, non-significant differences (paired *t* = 0.190, *P* = 0.851) between the *R* ^2^ values calculated using the two data sets (_T_ and _U_).

BA_T_ and BA_U_ were the response variables for which the best descriptive models were obtained (Table 4). For the best two-texture variable models, *R* ^2^ values were 0.93 and 0.92 for BA_T_ and BA_U_, respectively. These vegetation attributes had an *R* ^2^ value of 0.96 for the best three-texture variable models. Conversely, *R* ^2^ for the best descriptive model with one textural variable was 0.82. Unlike most vegetation variables, the best-fit models for Dn had much lower *R* ^2^ values, and these did not differ significantly from the values derived from the null model. The same occurred for the best-fit models for *H*' and D indices, both for the entire community and for the upper canopy, in the case of the three texture-variable models.

Comparison between the three types of models revealed that the most complex models did not necessarily incorporate the same variables as simpler models (Table 4). Most descriptive one-variable models included RED_VAR_ or RED_MEAN_ as the best explanatory variables of the behavior of vegetation variables (See Methods and Table 3 for a full description of texture variables and their abbreviations); NDVI_CORR_ and NDVI_SM_ were important only for Dn_U_ and Dn_T_. For two-variable models, either RED_VAR_ or RED_MEAN_ were retained in six models only, whereas variables incorporating textural information derived from vegetation indices (NDVI and EVI) became prominent. When moving to three-texture variable models, IR_MEAN_ emerged in eight models as capable of making a significant contribution to the descriptive power of the models. Conversely, in this set RED_MEAN_ and other RED-related textural variables became much less important, which indicates their limited descriptive ability in the presence of other textural variables.

The signs of the coefficients of the textural variables in the models changed according to the way in which they relate to the different response variables. Within the group of one-variable models, *D*_T_ and *D*_U_ were the only vegetation variables whose models had positive coefficients associated to the textural variables. This relation is less obvious for two- and three-texture variable models, yet the coefficients of the textural variables still have different sign when involved in the descriptive modeling of dominance as opposed to other vegetation variables.

### Predictive models of successional vegetation attributes

Most (27 out of 28 models) one- or two-texture variable best-fit models predicting vegetation response variables were identical to the respective descriptive models regarding the identity of the explanatory variables (see Table S2). Moreover, Spearman correlations between all possible *R* ^2^ and cross-validation *R* ^2^ (*R* ^2^_CV_) pairs of values for each vegetation variable and group of texture variables were very high. These correlations, which were higher for the one-texture variable models, were inversely related to the number of texture variables involved (Table 5). In general, *R* ^2^_CV_ values were lower than *R* ^2^ values in descriptive models, as low as 25%, but more often around 10% lower (Fig. 2, Table 4). Despite such reduction, BA_U_ and BA_T_ had *R* ^2^_CV_>0.90 in three-texture variable models. As was the case with descriptive models, there were no significant differences between the *R* ^2^ values of predictive models developed from the upper canopy and total sets (paired *t* = 0.634, *P* = 0.534).

**Table 5:** Spearman's *ρ* between descriptive *R* ^2^ and predictive *R* ^2^_CV_ values calculated for all linear models resulting from modeling each vegetation attribute as a function of one, two and three textural variables (TV).

TV	Age	Hgt	S_T_	S_U_	Dn_T_	Dn_U_	BA_T_	BA_U_	CC_T_	CC_U_	*H'*_T_	*H'*_U_	*D'*_T_	*D'*_U_
1	0.734	0.687	0.928	0.946	0.820	0.481	0.904	0.902	0.862	0.864	0.769	0.952	0.904	0.933
2	0.617	0.610	0.768	0.874	0.624	0.460	0.774	0.753	0.804	0.791	0.612	0.863	0.703	0.854
3	0.592	0.611	0.722	0.815	0.568	0.469	0.751	0.731	0.773	0.779	0.581	0.812	0.658	0.796

See Table 1 for vegetational attributes abbreviations and Methods for explanation of *R* ^2^_CV_ calculation.

**Figure 3:**
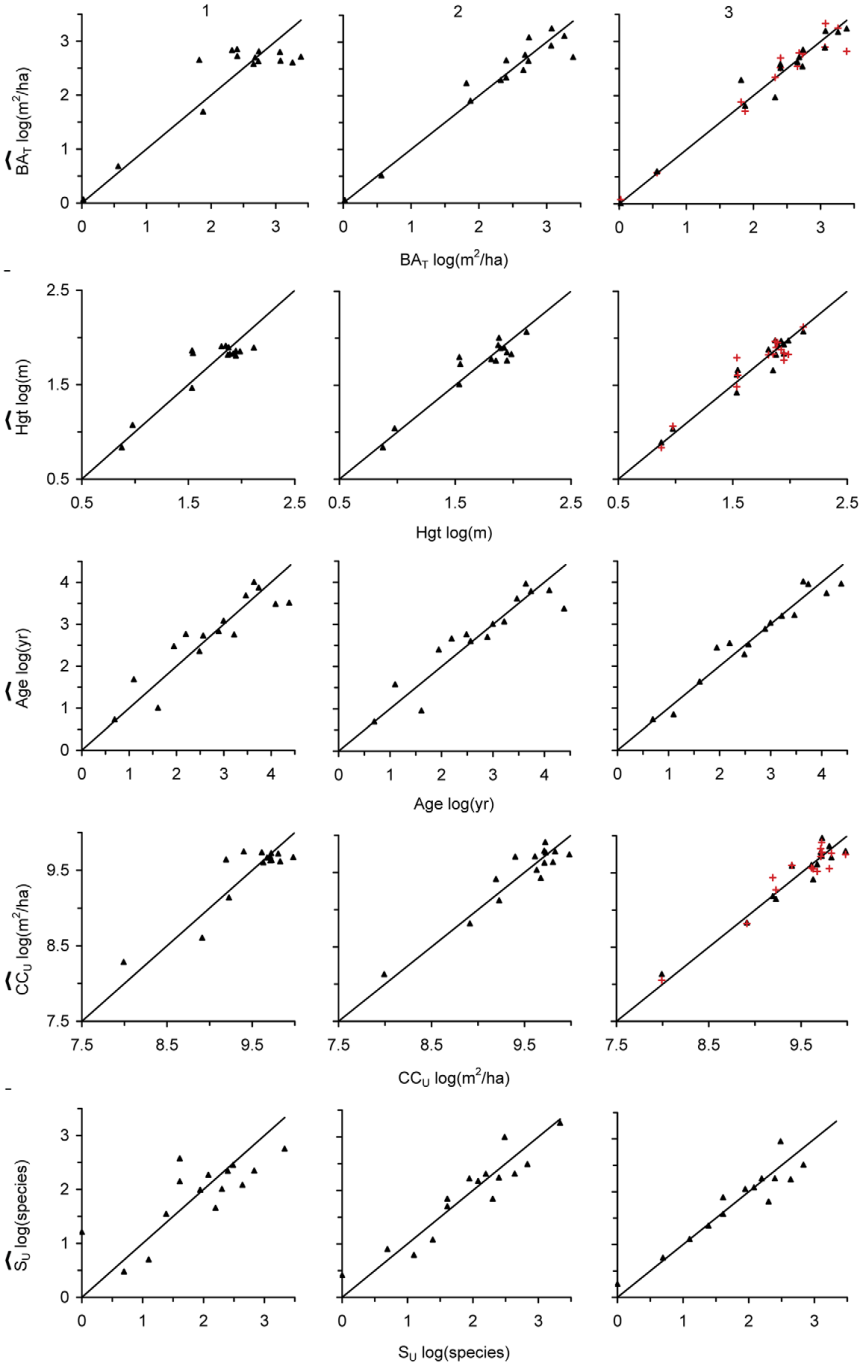
Observed (x-axes) vs. estimated (y-axes) values for the best descriptive (▴) and predictive (red +) linear models for vegetation attributes. See Table 1 for vegetational attributes abbreviations. Digits 1, 2, and 3 refer to the number of textural variables included in the model as explanatory variables.

Departure of predictive models from descriptive ones occurred mostly within the set of three-texture variable models (Fig. 3, Table S2). For the new set of predictive models, textural variables derived from the RED band (i.e. RED_VAR_ or RED_MEAN_) became prominent again among models with high *R* ^2^_CV_.

## Discussion

### Predictive potential of satellite image texture

In this study we demonstrate the large potential of image texture for predicting vegetation attributes during tropical forest succession. Texture is an emergent property of satellite images that is related to the neighborhood relationships among pixels [59], and thus it is capable of reflecting the internal organization (i.e. heterogeneity, directionality, entropy) of a region of interest, rather than on its mean properties. This seems to be the reason why the performance of texture-based analyses tends to exceed those based on spectral information in discriminating different successional stages [33], [46], [56]–[58], [62], [85]–[87]. High *R* ^2^ values comparable to those obtained by us have been reported by some studies, but only after complex image processing and modeling protocols based on mean canopy reflectance [30], [45], [73], [88]–[90]. Apparently this complexity has limited the broad application of these procedures, hence motivating the ongoing search for simpler solutions that are useful in a variety of circumstances. The method proposed in this study contrasts by its simplicity: the analysis was performed with a single image, and the models were linear and included few variables. Moreover, textural information can presently be extracted with ease. This simplicity, which becomes an asset in studying secondary vegetation and its attributes, depends on the basic principle that image texture actually reflects the internal heterogeneity of successional vegetation at the proper scale [62].

The ability to predict characteristics of secondary vegetation accurately depends on a combination of three relevant methodological aspects, all of which synergically contribute to the high predictive value of the models.

The first aspect is high image resolution. Typically, scholars interested in predicting vegetation attributes from space have used images with pixel size ≥30 m [28], [73], [88], [89], [91]. Agricultural fields derived from non-mechanized practices in tropical dry regions often have relatively small sizes (in our study area, the sizes of most successional stands range from 900 to 2,500 m^2^); thus a single such large pixel covers just one secondary vegetation stand. Therefore, high spatial resolution is required to detect and analyze the internal spatial variation typical of each secondary stand. Proisy et al. [33] came to a similar conclusion while mapping biomass in successional mangrove communities.

The second aspect was the inclusion of stand-level heterogeneity, an essential feature of successional vegetation. This inclusion was achieved by using a range of image textural attributes, some of which proved to have a very high predictive potential, even though our study also shows that many textural attributes do not have such potential (Table 4 shows nine variables that were included in only one model, in addition to 17 variables that were not included in any of them), in agreement with other studies [60], [62].

The third aspect was the decision to assess and contrast two large sets of models derived from alternative modeling procedures: one set included a limited number of descriptive models that included all sampling sites, whereas the other consisted of numerous predictive models constructed through leave-two-out cross-validation. The high degree of consistency between the models selected from either procedure confers increased reliability to the results, and implies that constructing descriptive models may suffice for assessing the secondary vegetation in a region. This is a valuable result as the construction of predictive models may require a large computational capacity as well as ample programming and statistical skills.

It is not uncommon for this kind of studies to face a limitation derived from the high cost of obtaining field information for every vegetation stand; thus having a large sample size, which would increase the accuracy in the predictions of the models, may not be feasible. This limitation was a strong motivation for this investigation. One would expect the prediction of vegetation attributes for new plots using our models to be flawed in two cases, neither of which occurred in our study. The first case would be if the models were used to estimate the attributes of plots with ages beyond those used in model fitting (extrapolation). We did not need to extrapolate because our plots represented the broadest possible successional gradient. The second case would be if the model's estimated coefficients were inaccurate due to a small sample size. We avoided this problem by using the leave-two-out cross-validation procedure [82]. Our high predictive *R* ^2^_CV_ values confirm that even a model based on a rarified sample was capable of providing reliable estimates for the vegetation attributes of new sites and warrants that our conclusions are not the artificial result of a small sample size.

An unanticipated conclusion from our study is that two-texture variable models should be preferred over three variable ones for describing and predicting vegetational attributes from image texture. This conclusion derives from two different results. On the one hand, AICc indicated that three-texture variable models were better than those with two variables only for half of the vegetational attributes, whereas for the other half both types of models were equally good. On the other, as the number of variables included in the models increased, the departure between observed and null *R* ^2^ distributions decreased, rendering three-variable models less reliable than two-variable ones. This conclusion is also relevant from a practical perspective, as the construction and validation of three-texture variable models requires much larger computing time and costs.

Despite previous suggestions that forest structure and diversity characteristics are preferably predicted from canopy-reflectance information [20], [73], in our case restricting the analysis to the upper canopy did not necessarily result in a better predictive capacity. In fact, our models predicting BA_T_ had higher *R* ^2^ values than BA_U_. In the case of an analysis based on texture of VHR imagery, predicting total community or upper canopy attributes can be done with comparable accuracy.

Even though we were able to demonstrate a high potential of GLCM textural indices to predict successional vegetation attributes, some caution must be exerted in using them. Like other indices, GLCM face potential important limitations that must be acknowledged. A particularly worrisome one is the fact that texture may be sensitive to image sun-view acquisition conditions [56]. Recently, Barbier et al. [92] proposed a mitigation method for FOTO (Fourier Transform Textural Ordination) indices that seems promising, albeit expensive and not totally straightforward. Further research is required aimed to develop a similar procedure to GLCM indices.

### The significance of image textural information

Understanding why some textural attributes are more useful than others in predicting vegetation properties, and therefore why they were repeatedly incorporated into the models, is important for a number of reasons. From a practical perspective this knowledge will orient future efforts to assess secondary vegetation by guiding researchers as to which variables they should focus on. Also, this information will provide a firmer ground for theoretical inquiry, as it represents an efficient way to identify relevant biological properties of the vegetation system and its spatially explicit spectral expression.

In this study it became clear that the three textural attributes that excelled in their predictive capabilities were IR_MEAN_, RED_VAR_ and RED_MEAN_ (Table 4), which indicates that in the context of texture, the predictive potential of the raw information contained in these bands exceeds that of NDVI and EVI, both based on RED and IR [47]. This implies that in calculating these indices the relevant spatial information that reflects the internal heterogeneity is lost. Under an approach centered on the examination of the internal heterogeneity of successional stands such loss of information is crucial at this scale of analysis, as objects that are not well differentiated spectrally can be finely discerned [20].

One finding that deserves particular attention is the inverse relationship between satellite-sensed heterogeneity, in particular the mean and the variance of textural variables, and ground-level vegetation development (i.e. stand age and other vegetation attributes). Large mean values obtained from a GLCM denote high levels of between-pixel spectral heterogeneity. Likewise, large GLCM variances indicate that such changes are highly variable regardless of the mean change. Therefore, although the inverse texture/vegetation relationship may seem counterintuitive and even contradictory to some recent findings reported in the literature [93]–[96], the explanation might lie in comprehending what the satellite actually perceives. One conceivable explanation is that the pixels corresponding to an early successional stand not only contain the reflectance properties of the plants, but also the spectral properties of the substrate on which they grow. If this interpretation is correct, it follows that textural attributes should show decreasing trends as vegetation structure becomes more complex and covers the soil. Thus the internal heterogeneity of a mature successional stand would be mostly related to the differences between less contrasting reflectance properties of the plants.

Our research is in agreement with other studies that have shown texture of satellite imagery to be closely related to the heterogeneity of the vegetation stand [57], [58]. For example, Frazer et al. [97] reported that LiDAR-derived indices such as lacunarity (the degree to which an object departs from a geometric pattern) are sensitive to canopy structure attributes. Despite the obvious ability of GLCM indices to reflect community-level attributes (e.g. total basal area, stand age), it is not yet clear how they relate to individual-level or other finer-scale traits (e.g. crown size). Thus, a promising line of future research will consist in finding out how these textural metrics relate to fine-scale vegetation and overall stand properties.

### Potential applications of image-texture-based modeling

The application of the method described here may produce important information related to two of the most relevant threats to biosphere integrity: climate change and biodiversity loss [98]–[101]. Basal area, the vegetation variable that was best predicted from image textural attributes, is strongly correlated with the standing biomass of a forest community [102], and thus to carbon storage [103]. Carbon sequestration rates may also be obtained by considering stand age [36], another variable accurately predicted from image texture. Therefore, by applying this procedure, it should be possible to assess and map with high confidence the spatial distribution of the potential carbon storage and sequestration in regions dominated by secondary vegetation in different stages of development.

Biodiversity conservation is one of the major goals of tropical ecologists nowadays [104]–[106]. Several efforts have been made recently to assess the possibility that local floras and faunas may persist in regions where native vegetation has undergone major transformations [14], [107]–[109]. Therefore, the possibility to predict species richness is of utmost importance. Our results show that species richness can be predicted with a precision close to 80%. This figure implies the existence of a relationship between the occurrence of different species in the terrain and the information sensed by a satellite. At present this topic is receiving much attention from researchers [55], [65], [110], [111], and our analysis opens new avenues to pursue it.

Canopy cover and vegetation height were also well predicted by our models. Again, there are several potential applications of this result. For example, information on canopy cover in a region dominated by secondary vegetation may help in assessing the potential soil erosion due to the kinetic energy of rainfall [112]–[114]. Similarly, it will provide information that can be used to assess habitat quality for a regional fauna, particularly for those animals whose survival depends on a closed canopy [115]–[117].

### Concluding remarks

Analyzing the extent and complexity of secondary vegetation by recognizing the spatial variation of its spectral information opens new and attractive research avenues; these differ substantially from previous efforts to study secondary vegetation that have been primarily based on the examination of spectral reflectance properties. Overall, the procedure is potentially usable in any successional plant community whose development involves large changes in heterogeneity through time. The current availability of VHR imagery, together with increasing computing capabilities, make it possible to develop faster and more efficient ways to assess the amount and condition of secondary vegetation in increasingly human-impacted regions world-wide based on this approach.

## Supporting Information

Table S1 **Within-site variability for vegetational variables.** See Table 1 for vegetational attributes abbreviations and units of measurement. SE: Standard error. M: mature forest.
(XLS)

Table S2 **Best descriptive and predictive linear models for vegetation attributes as a function of one, two and three textural variables (TV).** See Table 1 for vegetational attributes abbreviations. IR: near infra-red band, RED: red band, NDVI: Normalized Difference Vegetation Index, EVI: Enhanced Vegetation Index. See Table 3 for the description of textural variables denoted by subindexed terms MEAN, VAR, ASM, CORR, DR, SKEW, DISS, CONT, ENT, and HOM. Only those best predictive models that differed from the best descriptive ones are presented.
(XLS)
